# Clinical-demographic markers for improving diabetes mellitus diagnosis in people with tuberculosis in Tanzania

**DOI:** 10.1186/s12879-022-07249-x

**Published:** 2022-03-16

**Authors:** Kenneth Cleophace Byashalira, Nyasatu Godfrey Chamba, Yosra Alkabab, Peter Masunga Mbelele, Nyanda Elias Ntinginya, Kaushik Laxmidas Ramaiya, Mohamed Zahir Alimohamed, Scott Kirkland Heysell, Blandina Theophil Mmbaga, Ib Christian Bygbjerg, Dirk Lund Christensen, Stellah George Mpagama, Troels Lillebaek, ADEPT Consortium, Kenneth Cleophace Byashalira, Nyasatu Godfrey Chamba, Yosra Alkabab, Peter Masunga Mbelele, Nyanda Elias Ntinginya, Kaushik Laxmidas Ramaiya, Mohamed Zahir Alimohamed, Scott Kirkland Heysell, Blandina Theophil Mmbaga, Ib Christian Bygbjerg, Dirk Lund Christensen, Stellah George Mpagama, Troels Lillebaek, Jan-Willem Affenaar

**Affiliations:** 1 Kilimanjaro Christian Medical University College, Moshi, United Republic of Tanzania; 2 Kibong’oto Infectious Diseases Hospital, Sanya Juu, P.O. Box: 12, Siha, Kilimanjaro United Republic of Tanzania; 3 Kilimanjaro Christian Medical Centre, Moshi, United Republic of Tanzania; 4 Kilimanjaro Clinical Research Institute, Moshi, United Republic of Tanzania; 5 Division of Infectious Diseases and International Health, University of Virginia, Charlottesville, USA; 6 National Institute of Medical Research, Mbeya Medical Research Centre, Mbeya, United Republic of Tanzania; 7 Shree Hindu Mandal Hospital, Dar es Salaam, United Republic of Tanzania; 8 Department of Haematology and Blood Transfusion, Muhimbili University of Health and Allied Sciences, Dar es Salaam, United Republic of Tanzania; 9 Division Global Health Section, Department of Public Health, University of Copenhagen, Copenhagen, Denmark; 10 International Reference Laboratory of Mycobacteriology, Statens Serum Institut, Copenhagen, Denmark

**Keywords:** Implementation, DM screening, TB patients, Clinical-demographic, Tanzania

## Abstract

**Background:**

Tuberculosis (TB) control is threatened by an increasing prevalence of diabetes mellitus (DM), particularly in endemic countries. Screening for DM is not routinely implemented in Tanzania; therefore, we aimed to screen for DM at TB diagnosis using clinical-demographic markers.

**Methods:**

Our cross-sectional study recruited TB patients who received anti-TB treatment between October 2019 and September 2020 at health care facilities in three regions from Tanzania. Patients were screened for DM using DM symptoms (polydipsia, polyphagia and polyuria) and random blood glucose (RBG) testing. Patients with a history of DM and those with no history of DM but an RBG ≥ 7.8 mmol/L had point-of-care glycated haemoglobin (HbA1c) testing, and were considered to have DM if HbA1c was ≥ 48 mmol/mol.

**Results:**

Of 1344 TB patients, the mean age was 41.0 (± 17.0) years, and 64.7% were male. A total of 1011 (75.2%) had pulmonary TB, and 133 (10.4%) had at least one DM symptom. Overall, the prevalence of DM was 7.8%, of which 36 (2.8%) TB patients with no history of DM were newly diagnosed with DM by RBG testing. TB/DM patients were older than those with only TB (50.0 ± 14.0 years vs 40.0 ± 17.0 years, p < 0.001). Patients with RBG ≥ 7.8 mmol/L were more likely to have pulmonary TB (p = 0.003), age ≥ 35 years (p = 0.018), and have at least one DM symptom (p < 0.001). There was a substantial agreement (Kappa = 0.74) between the on-site glucometer and point-of-care HbA1c tests in detecting DM range of hyperglycemia.

**Conclusion:**

The implementation of clinical-demographic markers and blood glucose screening identified the overall prevalence of DM and those at risk of DM in TB patients. Clinical-demographic markers are independent predictors for DM range hyperglycemia and highlight the importance of further diagnostic testing and early co-management of TB and DM.

## Introduction

Although tuberculosis (TB) incidence is decreasing worldwide, the rapid increase of diabetes mellitus (DM) prevalence, particularly in TB endemic settings, threatens to dismantle the gains achieved in TB control [1–3]. In 2019, the World Health Organization (WHO) estimated 10 million people had active TB disease [4]. In the same year, 15% of individuals with TB had DM [5, 6]. In sub-Sahara Africa, the burden of TB is estimated to be 2.5 million, of which dual TB/DM contributes 9.0% [7, 8]. There has been a reported a three-fold increased risk of developing active TB for individuals with DM compared to those without [9, 10], a dual TB/DM disease contributing to poor TB treatment outcomes including increased treatment failures and increased mortality [9, 11]. Additionally, the clinical presentation of TB symptoms is reportedly more frequently non-specific for dual TB/DM disease resulting in delayed presentation and/or recognition by providers for testing [2, 12]. Despite the 2011 WHO recommendation of bidirectional screening for both DM and TB, most TB endemic settings, including Tanzania [13], have not fully implemented this practice in clinical settings [14, 15]. Tanzania has adopted WHO guidance and developed a local guideline for collaborative TB and DM care activities, including recommendations for bidirectional screening of TB and DM epidemics [13], however potential barriers to practical implementation include a lack of training of health care providers, a lack of availability of DM diagnostic tools such as glucometers at TB clinics, and a lack of studied benefit of any implementation approach [5, 14–16]. For instance, a readiness assessment conducted in Tanzania found less than one-third of health care providers possessed skills for TB/DM diagnosis and co-management, and only 7% of 30 health facilities at various levels (health centres, districts hospitals and regional referral hospitals) had glucometers accessible for TB clinics. There were no organized bidirectional TB and DM services at any of the health care facilities [15, 17].

In response to the stark findings of the readiness assessment, we designed an Adaptive Disease control Expert Programme model in Tanzania (ADEPT) to guide the country to integrate DM screening and care into the TB health care services [18]. Our initial plan included integrating dual screening and linkage of TB and DM in the health system, and increasing the training of health care providers regarding DM screening using diagnostic equipment such as glucometers with gluco-strips and glycated haemoglobin (HbA1c) at various levels of health facilities [18]. The aim was to perform DM screening at the TB clinic according to the Ministry of Health, Community Development, Gender, Elderly and Children’s national guideline for TB/DM collaborative care [13].

However, Tanzania is a low-middle income country, with the majority of people with TB living in considerable poverty [15]. Systems of TB care are guided by restricted budgets and the allocation of resources determined well in advance of distribution [19]. Strategies to more effectively implement a TB/DM diagnostic and management guideline are therefore of utmost importance. The current algorithm for TB/DM diagnosis has not been formally studied in Tanzania and is somewhat based on extrapolation from other recommendations on the topic. Additionally, the current algorithm does not include steps for potential triage, such as questions related to the classical symptoms of advanced DM disease, polyphagia, polydipsia, and polyuria that signify intracellular glucose deficiency [20]. Furthermore, there are no age-specific recommendations for DM screening among active TB patients in Tanzania, as suggested in other countries such as India, to increase the detection of DM in older age individuals in whom DM is more common [21]. We, therefore, sought to assess the feasibility and yield of DM screening in active TB patients across all health facilities in three different geographic regions in Tanzania based on other clinical and demographic characteristics for predicting DM range hyperglycaemia.

## Methods

### Study settings

The study was implemented in health facilities located in three regions in Tanzania: Dar es Salaam, Iringa and Kilimanjaro. Dar es Salaam is the largest city located in the Eastern zone of Tanzania, with an estimated population of 4.4 million inhabitants by the 2012 census [22] and an annual growth rate of 5.6% [23]. Dar es Salaam is the major contributor to TB incidence, with 18% of the new TB patients diagnosed annually [24]. Iringa in southern-central Tanzania has the 2nd highest TB incidence due to a higher HIV/AIDS prevalence rate. Kilimanjaro, located in northern Tanzania, has a high prevalence of glucose impairment in the general population of 21.7% [25], but importantly Kilimanjaro borders with Kenya to the north and supports migrant populations with different access to healthcare services.

### Study design and population

The study employed a cross-sectional design to screen for DM in patients with active TB (both newly diagnosed TB and patients receiving TB treatment). The TB patients were defined based on the National Tuberculosis and Leprosy Program guideline in Tanzania. In each region, all TB patients of all forms, diagnosed from October 2019 to September 2020 (1 year), were included in our study. A total of 32 health facilities [regional referral hospitals (n = 3), district hospitals (n = 7) and health centres (n = 22)] offering TB diagnosis and treatment, with or without DM services, were systematically selected for DM screening procedures. The study was granted ethical approval by the Kilimanjaro Christian Medical University College (Ref No. 2482) and the National Institute for Medical Research in Tanzania (NIMR/HQ/R.8a/Vol.IX/2988). Further permission to conduct this study was approved by the relevant institutional review board and local authorities in Dar es Salaam, Iringa and Kilimanjaro regions.

ADEPT is a programme, which aims to strengthen the health system in managing communicable and non-communicable diseases using TB/DM as a case study in Tanzania. Before the implementation, we used a stepwise training approach for knowledge and skills improvement for the health care providers as described in the ADEPT model [18]. In brief, health care providers (medical doctors and nurse officers) from the implementation sites were trained using both web-based and face-to-face modules to acquire theoretical and clinical skills on the objectives, methodology and procedures. At the same time, their competencies are being accessed using predefined criteria [18]. Additionally, each of the health facilities was issued a diabetes screening kit containing; a POC glucometer machine with glucose test strips (GlucoPlus™ Inc; 2323 Halpern, Ville St-Laurent, Canada), and HbA1c analyser (HemoCue Hb1c 501 system- HemoCue AB;SE-262 23, Ängelholm, Sweden), a portable point of care device which is not affected by TB induced hyperglycaemia, and does not require a fasting state [2] with HbA1c cartridges. Printed screening algorithms also guided health care providers for DM screening in patients with TB. All the tools were free of charge supplied by the ADEPT programme.

### DM screening procedures in patients with TB

All registered TB patients were asked if they had a known history of DM and were prescribed DM medications. In patients with a known history of DM, medical charts were reviewed to confirm the prescribed DM medications, followed by a point of care (POC) HbA1c testing to assess their glycaemic severity regardless of whether they had been prescribed DM medications. Patients were then provided context-tailored education about glycemic control and adherence strategies for TB and DM medications.

TB patients with no history of DM were asked about the presence or absence of any DM symptoms: polyphagia, polydipsia and polyuria [2], followed by random blood glucose (RBG) testing regardless of the presence or absence of DM symptoms, using the POC glucometer machine. We classified patients’ blood glucose levels as normal (RBG < 7.8 mmol/L), pre-DM (RBG > 7.8–11.0 mmo/L) and DM (RBG ≥ 11.1 mmol/L) as per the International Diabetes Federation and the national TB/DM guidelines [20]. Next, TB patients without a known history of DM who had RBG levels in either the pre-DM or DM range were offered POC HbA1c testing. Validation of the HbA1c test was performed using control samples. Venous blood samples (4 µL) were collected using EDTA tubes and analysed within 5 min. HbA1c results were reported as mmol/mol and interpreted as < 39 mmol/mol (normal), 39 to < 48 mmol/mol (pre-DM) and ≥ 48 mmol/mol as (DM) [2].

### Data management and statistical analysis

A standardized data sheet was used to collect patients’ demographics and clinical information. De-identified data and appropriate statistical methods were analysed with the IBM SPSS version 24 (IBM SPSS, Armonk, NY, USA). Mean with standard deviation (SD) or median with the 25th and 75th interquartile range (IQR) were used to summarize parametric and non-parametric continuous variables. Comparison of categorical variables such as frequencies and proportions of participants screened and diagnosed for normoglycaemia, hyperglycaemia (pre-DM or DM) were performed using the Pearson Chi-square or Fisher’s exact test. Independent *t-*test and Mann–Whitney U-tests were used to compare parametric and non-parametric continuous variables, including age, weight, RBG, and HbA1c levels, respectively. A multivariate logistic regression analysis was performed to calculate the odds ratio (OR) with 95% confidence intervals (CI) for risk factors that predicted DM range RBG levels in patients without a history of DM. A p-value of < 0.05 was considered statistically significant.

## Results

### Baseline characteristics of study participants

**Fig. 1:**
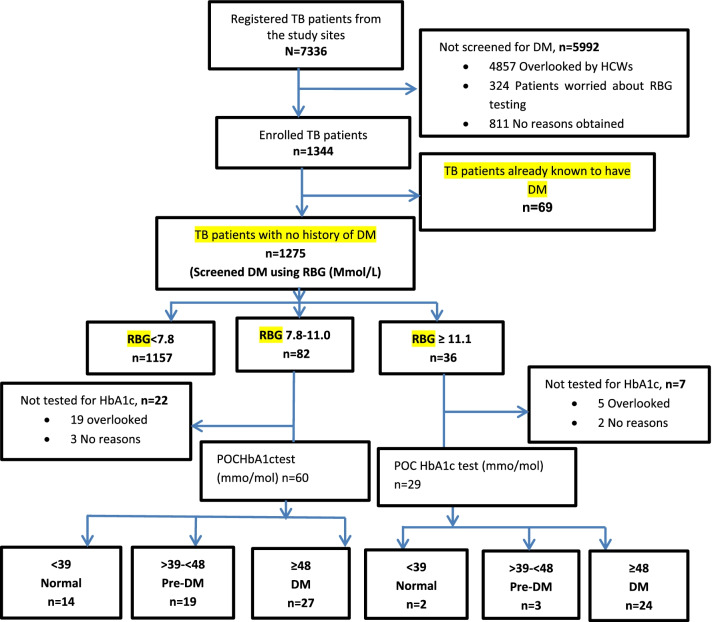
Flow chart for diabetes mellitus screening in patients with tuberculosis. TB: tuberculosis, DM: diabetes mellitus, HCW: health care workers, RBG: random blood glucose, POC HbA1c: point-of-care glycated haemoglobin

7336 participants were registered at TB clinics from all recruitment sites during the study period (Fig. 1), including 4359 TB patients registered in Dar es Salaam study sites and 1384 TB patients from Iringa sites and 1593 patients in Kilimanjaro sites. Overall, DM screening from all the implementation sites was performed in 1344 (18.3%) TB patients and was included for the final analysis. Dar es Salaam region screened for DM in 744 (17.0%) of total TB patients registered in the area, Iringa screened 330 (23.8%), and Kilimanjaro 220 (13.8%).

Of the 1344 participants screened for DM, 870 (64.7%) were men, and the mean age was 41 (± 17) years (Table 1). 1303 (96.9%) had no previous history of TB, 680 (50.6%) had bacteriologically positive TB disease diagnosed by Xpert MTB/RIF and/or sputum smear microscopy tests, and 335 (25%) were living with HIV, of which 308 (91.9%) were on antiretroviral therapy (ART). Furthermore, 69 (5.1%) TB patients reported a history of DM and were older compared to TB patients without a history of DM (50.0 ± 15.0 versus 41.0 ± 17.0 years, p < 0.001).

**Table 1:** Characteristics of study participants with and without a history of diabetes (N = 1344)

Characteristics	Total N = 1344 (%)	History of DM N = 69 (%)	No history of DM N = 1275 (%)	p-value^a^
Mean age (± SD) year		50 (± 15)	41 (± 17)	< 0.001
Mean weight (± SD) kg		61 (± 14)	52 (± 13)	< 0.001
Sex				
Female	474 (35.3)	31 (44.9)	443 (34.7)	0.085
Male	870 (64.7)	38 (55.1)	832 (65.3)	
HIV status (n = 1342)^a^				
Positive	335 (25.0)	18 (26.5)	317 (24.9)	0.774
Negative	1007 (75.0)	50 (73.5)	957 (75.1)	
On ART (n = 335)				
Yes	308 (91.9)	15 (83.3)	293 (92.4)	0.168
No	27 (8.1)	3 (16.7)	24 (7.6)	
Location of TB disease				
PTB	1011 (75.2)	53 (76.8)	958(75.1)	0.754
EPTB	333 (24.8)	16 (23.2)	317 (24.9)	
Bacteriological results^b^				
Positive	680 (50.6)	34 (49.3)	646 (50.7)	0.822
Negative	664 (49.4)	35 (50.7)	626 (49.3)	
TB history				
New TB	1303 (96.9)	66 (95.7)	1237 (97.0)	0.464
Recurrent TB	41 (3.1)	3 (4.3)	38 (3.0)	

TB: tuberculosis DM: diabetes mellitus, SD: standard deviation, PTB: pulmonary tuberculosis, EPTB: Extra-pulmonary tuberculosis, HIV: human immunodeficiency virus, ART: antiretroviral therapy

^a^Two participants had unknown HIV status, a = x^2^ test

^b^Diagnosed by Xpert MTB/RIF and/or sputum smear tests

### Characteristics of TB participants without prior history of DM screened for diabetes using RBG levels

The overall DM prevalence in 1344 participants was 7.8% (n = 105), including 36 (2.8%) with newly diagnosed DM patients by RBG testing. Table 2 summarizes the clinical characteristics of 1275 TB patients without a known history of DM who were screened for DM symptoms, followed by RBG, and then HbA1c tests if the RBG was in the pre-DM or DM range. All 1275 had both symptom testing and RBG testing. Overall, 133 (10.4%) of the 1275 TB patients had at least one DM symptom, including polydipsia in 51 (4%), polyphagia 72 (5.6%) and polyuria in 99 (7.8%). RBG diagnosed DM and pre-DM range hyperglycaemia in 36 (2.8%) and 82 (6.4%) patients, respectively. Of the people newly diagnosed with DM, Dar es Salaam region contributed 50.0%, Iringa and Kilimanjaro regions each contributed 25.0% of new DM cases. Furthermore, the sites in Dar es Salaam region, Iringa region, and Kilimanjaro region diagnosed DM in 18 (2.6%), 9 (2.8%), and 9 (4.4%) of the total TB patients screened for DM, respectively.

**Table 2:** Characteristics of tuberculosis participants screened for diabetes mellitus by random blood glucose excluding previously known diabetic patients (N = 1275)

Characteristics	DM (≥ 11.1) N = 36 (%)	p-value	Pre-DM (7.8–11.0) N = 82 (%)	Normal (< 7.8) N = 1157 (%) (Reference)	p-value
Mean age (± SD) year	50 (± 14)	0.001	46 (± 18)	40 (± 17)	0.001
Mean weight (± SD) kg	61 (± 16)	0.001	55(± 15)	51 (± 13)	0.064
Sex					
Female	15 (41.7)	0.376	18 (22.0)	410 (35.4)	0.011
Male	21 (58.3)		64 (78.0)	747 (64.6)	
HIV status					
Positive	8 (22.2)	0.710	18 (22.0)	291 (25.2)	0.518
Negative	28 (77.8)		64 (78.0)	866 (74.8)	
On ART (n = 317)					
Yes	8 (100)	0.473	14 (77.8)	267 (91.8)	0.063*
No	0 (0.0)		4 (22.2)	24 (8.2)	
Type of TB					
PTB	28 (77.8)	0.710	68 (82.9)	862 (74.5)	0.095
EPTB	8 (22.2)		14 (17.1)	295 ( 74.5)	
TB history					
New TB	35 (97.2)	0.942	80 (97.6)	1122 (97.0)	0.551*
Recurrent TB	1 (2.8)		2 (2.4)	35 (3.0)	
Bacteriological status					
Positive	17 (47.2)	0.675	47 (57.3)	582 (50.3)	0.219
Negative	19 (52.8)		35 (42.7)	575 (49.7)	
DM symptoms					
Polydipsia (Yes, n = 51)	12 (33.3)	< 0.001	10 (12.2)	29 (2.5)	< 0.001
Polyphagia (Yes, n = 72)	13 (36.1)	< 0.001	24 (29.3)	35 (3.0)	< 0.001
Polyuria (Yes, n = 99)	18 (50.0)	< 0.001	32 (39.0)	49 (4.2)	< 0.001
≥ One DM symptoms					
Yes	22 (61.1)	< 0.001	37 (45.1)	74 (6.4)	< 0.001
No	14 (38.9)		45 (54.9)	1083 (93.6)	

RBG: Random blood glucose

^a^Fishers Exact test was used

The mean age of participants with DM range glycaemia by RBG was (50.0 ± 14.0) years, and their weight was significantly higher (61.0 ± 16.0 kg) compared to TB patients with normal RBG levels (51.0 ± 13.0 kg) (p = 0.001). The proportion of pre-DM was significantly higher in men (78%) versus women (12%) compared to those with normoglycaemia (p = 0.011). Still, these differences were not significant when comparing those with DM range hyperglycaemia and normoglycaemia. Eighty-nine (75.4%) participants with DM and pre-DM were assessed for glycaemic severity using the HbA1c test (Fig. 1). There was a substantial agreement between glucometer and HbA1c tests (Kappa = 0.74).

### Risk factors associated with high RBG levels in TB patients

In multivariate regression of patients without a known history of DM, TB patients who presented with at least one of the DM symptoms (OR 23.07, 95% CI 13.94–39.09, p < 0.001) had an age of ≥ 35 years (OR 2.16, 95% CI 1.35–3.55, p = 0.018), or had pulmonary TB disease only (OR 3.05, 95% CI 1.57–6.09, p = 0.003) were significantly more likely to have RBG in the DM range (Table 3).

**Table 3:** Multivariate analysis to assess factors associated with high RBG levels among TB patients without a known history of DM (N = 1275)

Variables	Total N = 1275	Unadjusted OR (95% CI)	p-value	Adjusted** OR (95% CI)	p-value
Sex					
Female	44 (34.7)	Reference			
Male	832 (65.3)	1.41 (0.93–2.15)	0.106	2.21 (1.33–3.70)	0.002
Age group					
< 35	490 (38.5)	Reference			
≥ 35	785 (61.5)	2.14 (1.38–3.33)	0.001	2.16 (1.35–3.55)	0.018
≥ One DM symptoms					
No	1148 (89.6)	Reference			
Yes	133 (10.4)	14.64 (9.51–22.52)	< 0.001	23.07 (13.94–39.09)	< 0.001
HIV status					
Negative	958 (75.1)	Reference			
Positive	317 (24.9)	0.84 (0.53–1.33)	0.456	0.70 (0.41–1.17)	0.192
On ART					
No	28 (91.2)	Reference			
Yes	289 (8.8)	0.49 (0.16–1.55)	0.219		
Location of TB					
EPTB	317 (24.9)	Reference			
PTB	958 (75.1)	1.49 (0.92–2.42)	0.103	3.05 (1.57–6.09)	0.003
TB history					
New TB	38 (3.0)	Reference			
Recurrent TB	1237 (97.0)	1.20 (0.42–5.01)	0.769	1.06 (0.33–5.01)	0.925
Bacteriological results					
Negative	629 (49.3)	Reference			
Positive	646 (50.7)	1.17 (0.80–1.71)	0.416	1.09 (0.65–1.85)	0.741

OR: Odds ratio, CI: confidence interval

**Adjusted for sex, age, DM symptoms, HIV, type of TB, TB registration group, and bacteriological results. Logistic regression analyses were used

## Discussion

Our study examined the implementation of clinical-demographic markers and blood glucose screening to guide the identification of people at risk of DM among those presenting with active TB disease in three diverse regions in Tanzania. Our major findings were that among people without a known history of DM, the presence of classical symptoms of DM (polyphagia, polydipsia and polyuria), age ≥ 35 years, and exclusive pulmonary TB were significant predictors of DM range RBG. These subgroups may represent the highest yield for additional confirmatory testing, such as with HbA1c.

The overall DM prevalence of 7.8% among patients with active TB, including those with a known prior diagnosis of DM, was lower than other previous studies of people with TB performed in Dar es Salaam (9.7%) [26] and Mwanza (16.7%) [27] regions of Tanzania, but more than twice as high the estimated 3.2% of the general population in the country [28]. This variation in DM prevalence in our study is likely due to the differences in populations with TB previously tested and the methods of DM screening. For instance, participants included in the study by Faurholt-Jepsen et al. from Mwanza region-Tanzania had bacteriologically confirmed TB only and might thus have been at higher risk of DM compared to clinically diagnosed TB patients [2]. Our findings are in accordance with the DM prevalence among TB patients reported from China (7.7%) [29], but lower than the pooled sub-Saharan African report and other studies conducted on DM prevalence among TB patients [3, 6, 7, 30–33]. The variation in DM prevalence across these studies and ours might be explained by differences in sample size, population, local setting, TB burden across the regions, and methods used for DM diagnosis. For instance, the background rate of DM in the general population of India is much higher than in Tanzania and most of the African continent and likely explains the differences in screening yield in studies from the Indian subcontinent compared to those in sub-Saharan Africa [30, 34].

DM symptoms (polyuria, polyphagia and polydipsia) have been established as cardinal symptoms for presuming DM, particularly type 2 DM, in the general population [2]. Our study observed that only 10% of participants reported yes to at least one DM symptom at baseline, yet 61% of DM cases had at least one of these symptoms. Our study findings in the Tanzanian context importantly differ from other reports that have previously suggested that DM symptoms are non-specific to individuals with TB/DM [2, 9]. Our findings highlight context-specific screening of TB patients and the use of classical DM symptom screening as a relatively high-yield and low-cost starting point for further triage to another diagnostic testing. For instance, our study demonstrated that implementation of this algorithm increased the yield of DM detection in TB patients by an absolute 3%, and notably, those patients were rapidly given disease information and triaged to DM tailored management. These findings are in keeping with a report from Nigeria in which implementation of the algorithm not only increased DM diagnostic yield but also proved feasible and acceptable among health care providers [35].

Variations in contexts across epidemiological settings may be essential to generate local demo-clinical markers, which are cost-effective to both implementers and patients for optimal DM screening in patients with TB. Several studies have reported age above 40 years as a non-modifiable risk factor for DM [6, 29, 31, 35]. So it was expected that we observed that TB patients identified with DM range RBG or with a known history of DM were significantly older than those with TB alone, similar to other studies of DM screening among people with TB [36]. Our analyses used an age cut-off of ≥ 35 years to identify those at higher risk of DM range RBG. A similar observation was made in a study from Zambia, where the mean age of people with DM and TB was 33 years [37]. In contrast, studies from other regions with lower TB prevalence have noted much older subgroups with TB/DM [38], highlighting the importance of screening strategies based on the local epidemiological context [2].

Furthermore, our study observed that pulmonary TB was associated with a higher RBG range in TB patients compared to those patients with extra-pulmonary TB. This finding is consistent with other studies, which have found, on average, a threefold higher risk of DM in pulmonary TB patients compared to those with extra-pulmonary TB [31, 39, 40]. Other evidence has shown that TB patients with concurrent DM present clinically with more pulmonary cavities [41] than patients with TB alone, resulting in a higher sputum bacterial load [42].

Our study found that 26 (7.8%) TB/HIV co-infected participants had DM, and three of them were not on ARTs. A prior study in Dar es Salaam observed that TB patients with HIV not on ART had a higher risk of developing DM than those on ART [43]. Other studies of TB observed that those living with HIV had a reduced risk of DM compared to those without HIV [27, 44]. Nevertheless, dual TB/DM and TB/HIV are known independent risk factors for poor TB treatment outcomes [43, 45, 46]. Therefore, given our findings of distinct subpopulations with TB/DM and TB/DM/HIV, further implementation studies in Tanzania should focus on early linkage to and retention in collaborative and multidisciplinary care.

We noted important regional differences in the uptake of the screening procedures and yield of diagnosing DM range RBG. We observed that only 17% of the total TB patients registered in Dar es Salaam, 23% in Iringa, and 14% in Kilimanjaro were screened for DM during the study period. The lack of screening uptake occurred despite a refresher training conducted for health care providers on the procedures and inventory assurance for all necessary tools for DM screening. However, our findings of these initial efforts are in line with other studies which have observed low uptake of DM screening during early practice change [14, 31, 47]. Lower than expected uptake of DM screening in our study may be explained by the fact that we implemented the study during the COVID-19 pandemic, where more resources and personnel were occupied with COVID-19 response activities. Nevertheless, a focused review of the screening procedures with front-line health care workers toward screening practice improvement is underway and beyond the scope of the current discussion.

A major strength of the study is the systematic implementation across three demographically different regions in Tanzania and the use of both diagnostic testing with RBG and HbA1c for assessing RBG severity, as well as the use of DM clinical symptoms. Yet given the implementation in routine clinical settings, the study had several potential limitations. First, a smaller than expected percentage of TB patients were screened for DM, which raises the possibility of selection bias if healthcare providers preferentially perform screening in those suspected to have DM. Secondly, analysis of the risk factors was based on the RBG results and not on the HbA1c. RBG has a lower sensitivity than HbA1c [48], and there is the potential of missing DM patients who present with wasting disease secondary to TB disease and did not have a DM range RBG at presentation, which otherwise would have underestimated the true prevalence of DM. Furthermore, RBG and HbA1c testing at the time of TB diagnosis do not fully differentiate transient TB disease-induced hyperglycaemia that resolves with TB treatment alone and may falsely overestimate DM prevalence if glycaemia screening is not performed at the end of TB treatment.

We have yet to perform longer-term follow-up in patients with DM range RBG or HgbA1c to determine the persistence of the hyperglycaemia phenotype and to determine late-stage TB treatment outcomes or DM-related complications. Lastly, we did not have height measures, and therefore we could not calculate body mass index (kg/m^2^), which may be a better measure for relative anthropometric categories than weight alone to predict the risk of developing DM.

## Conclusion

Among patients with active TB in three representative regions in Tanzania, the overall prevalence of DM was 7.8%, and a further 6.4% were at high risk of DM based on pre-DM range RBG measurements at the time of TB diagnosis. Clinical-demographic markers such as at least one classic DM symptom, pulmonary TB, and age of 35 years or above were independent predictors for DM range RBG. These markers are recommended to prioritize further diagnostic testing and earlier co-management of hyperglycaemia and TB.

## Data Availability

Data used in this study are only available under restricted access through authors due to Tanzanian data protection legislation. However, data and materials used in this manuscript will be available on request from the corresponding author.

## Abbreviations

ADEPT: Adaptive Diseases Control Expert Program in Tanzania
ART: Antiretroviral therapy
CI: Confidence intervals
COVID: Coronavirus disease
DM: Diabetes mellitus
EDTA: Ethylenediamine tetraacetic acid
EPTB: Extra-pulmonary tuberculosis
HbA1c: Glycated haemoglobin
HIV: Human immunodeficiency virus
NIMR: Nation Institute for Medical Research
OR: Odds ratio
POC: Point of care
PTB: Pulmonary tuberculosis
RBG: Random blood glucose
SD: Standard deviation
SPSS: Statistical package for the social science

## Declarations

### Accordance statement

All methods were carried out in accordance with relevant guidelines and regulations.
